# An Unusual Case of Lung Adenocarcinoma Metastasis to the Tricep Muscle: Four Years Disease-Free After Surgical Resection and Radiotherapy

**DOI:** 10.7759/cureus.38347

**Published:** 2023-04-30

**Authors:** Prabasha Weeraddana, Thilini Walgamage, Ragaa Elkabbani, Mikhail Dmitriev, Juan Crespo-Quezada, Mehndi Dandwani, Wenli Gao

**Affiliations:** 1 Internal Medicine, Danbury Hospital, Danbury, USA; 2 Pathology, Danbury Hospital, Danbury, USA; 3 Internal Medicine, Connecticut Institute for Communities (CIFC), Danbury, USA; 4 Oncology, Danbury Hospital, Danbury, USA

**Keywords:** disease-free for four years, oligo metastasis, metastasis in skeletal muscles, metastasis to the trapezius muscle, adenocarcinoma lung

## Abstract

Lung cancer is one of the leading causes of cancer-related death worldwide. Lung cancer commonly metastasizes to the liver, bone, and brain, but metastasis to skeletal muscles is rare. The development of metastasis in skeletal muscles indicates stage IV disease with a poor prognosis. The most effective treatment strategy is unclear. Palliative radiotherapy is often used to treat skeletal muscle metastases, and patient survival is poor, with an average survival of one year. Here we discuss the case of a 76-year-old female diagnosed with lung adenocarcinoma with metastasis to the trapezius muscle. Initially, she was treated with stereotactic body radiotherapy for stage T1 lung adenocarcinoma. Her follow-up surveillance positron emission tomography (PET) scan in 11 months showed an abnormal focal area of increased activity localizing to the long head of the right triceps muscle. The diagnosis was confirmed with an ultrasound-guided biopsy of the trapezius muscle. Following that, the patient underwent wedge resection of the right middle and upper lobe of the lung and partial right trapezius resection. Afterward, she was given radiation therapy at the tricep resection site. She remained disease-free for four years after excision and radiation therapy.

## Introduction

Lung cancer is common cancer affecting patients worldwide and is a leading cause of a significant number of deaths. In 2018, 1.8 million people died from lung cancer, which accounts for 18.4% of cancer-related deaths [1]. Adenocarcinomas are the most common among non-small cell lung cancers (NSCLCs), with a prevalence ratio of 40%. Although NSCLC is known to metastasize, skeletal muscles are a rare site of metastasis and are reported to be less than 1% of all NSCLC metastasis sites [2]. Here, we report a rare case of lung adenocarcinoma with metastasis to the tricep muscle that was successfully treated with surgical resection and postoperative radiation. To our knowledge, this is the first case reported of lung adenocarcinoma with metastasis to the triceps muscle that remains disease free for four years following surgical resection and radiation therapy.

## Case presentation

**Figure 1:**
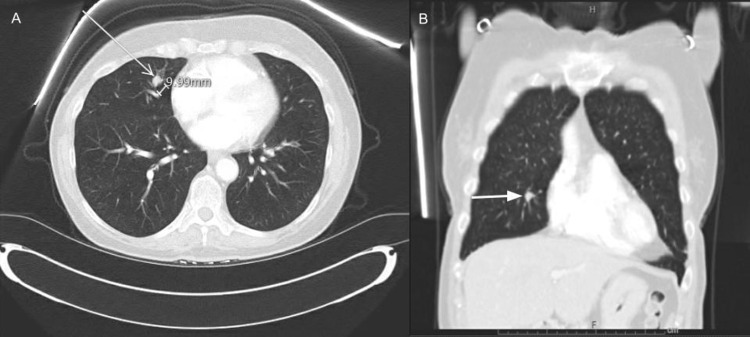
CT scan of the chest showed a right middle lobe pulmonary nodule approximately 9.99 mm in diameter concerning for malignancy (A: axial view, B: coronal view).

**Figure 2:**
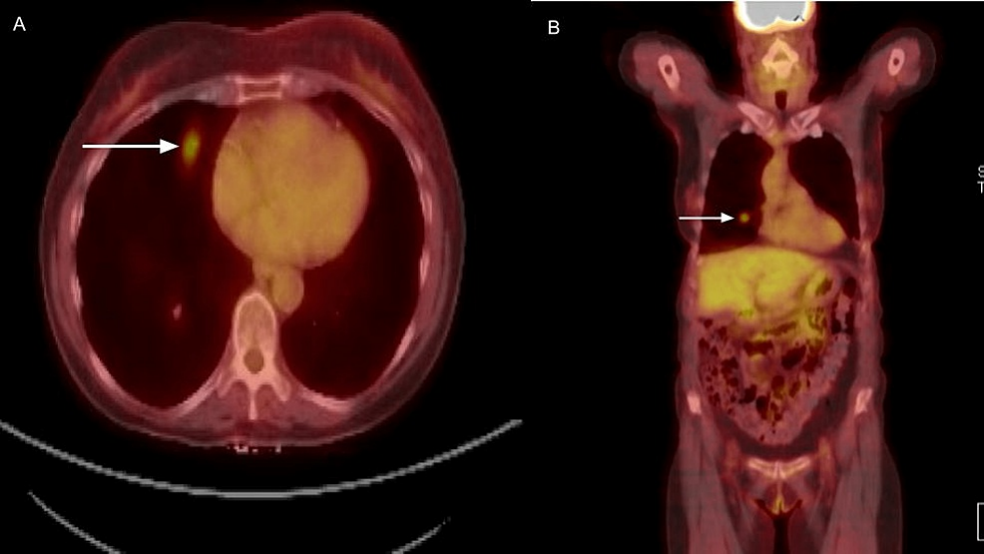
PET-CT showed focal activity with a standardized uptake value (SUV) maximum of 5.5 localizing to the suspicious right middle lobe lung nodule (A: axial view, B: coronal view). PET-CT: Positron emission tomography-computed tomography.

**Figure 3:**
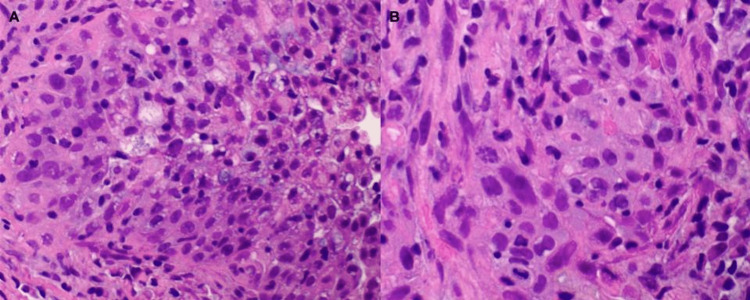
A right lung middle lobe biopsy demonstrated invasive, poorly differentiated adenocarcinoma. A) (H&E, x200) Poorly differentiated cells arranged in sheets with solid and micropapillary growth patterns characterized by a lack of fibrovascular core. B) At higher power (H&E, x400), the lack of glandular formation of the neoplastic cells is demonstrated.

A 76-year-old female with a history of stage IA (T1cN0) intracystic papillary carcinoma of the left breast after undergoing a lumpectomy followed up at the oncology clinic. She had a 40-pack-year smoking history and used alcohol occasionally. She denied any family history of malignancy. Following the lumpectomy, adjuvant radiation therapy was planned, and right hilar fullness was incidentally noted at the time of radiation therapy. A follow-up CT scan of the chest showed a right middle lobe pulmonary nodule approximately 1 cm in diameter concerning malignancy (Figure 1). Positron emission tomography-Computed tomography (PET-CT) showed focal activity with a standardized uptake value (SUV) maximum of 5.5 localizing to the suspicious right middle lobe lung nodule (Figure 2). CT-guided lung biopsy returned poorly differentiated invasive adenocarcinoma with solid and micropapillary growth patterns (Figure 3).

**Figure 4:**
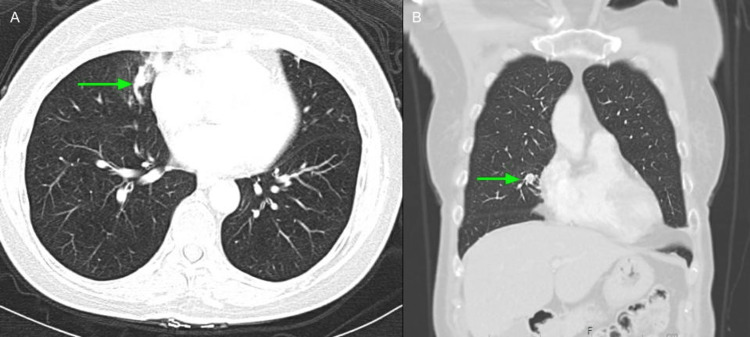
Chest CT at 11 months showed an interval increase in the right middle lobe nodule (green arrow) concerning tumor progression (A: axial view, B: coronal view).

**Figure 5:**
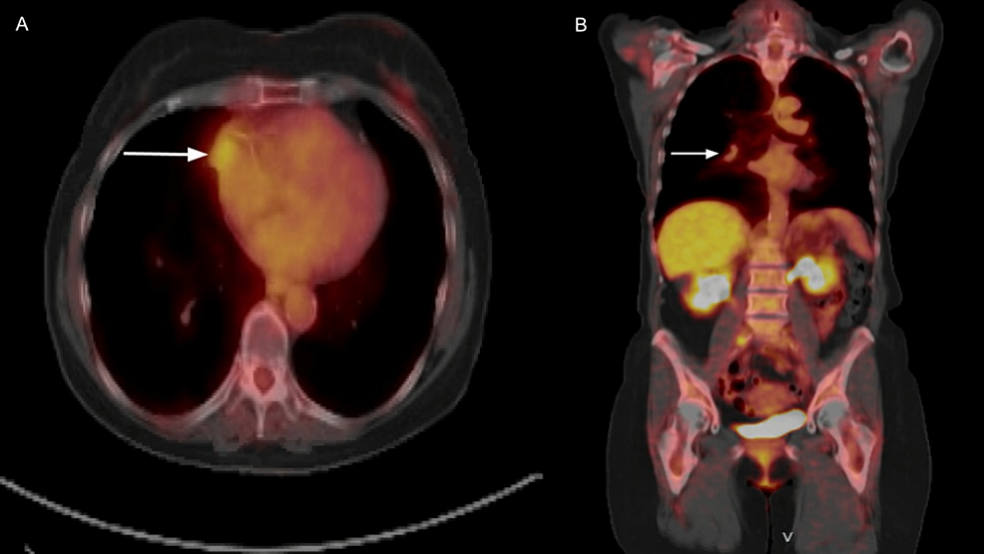
Follow-up PET-CT revealed mild activity (white arrows) in the right middle lobe (A: axial view, B: coronal view). Although the area appeared slightly larger relative to the prior, it showed low-grade activity. PET-CT: Positron emission tomography-computed tomography.

**Figure 6:**
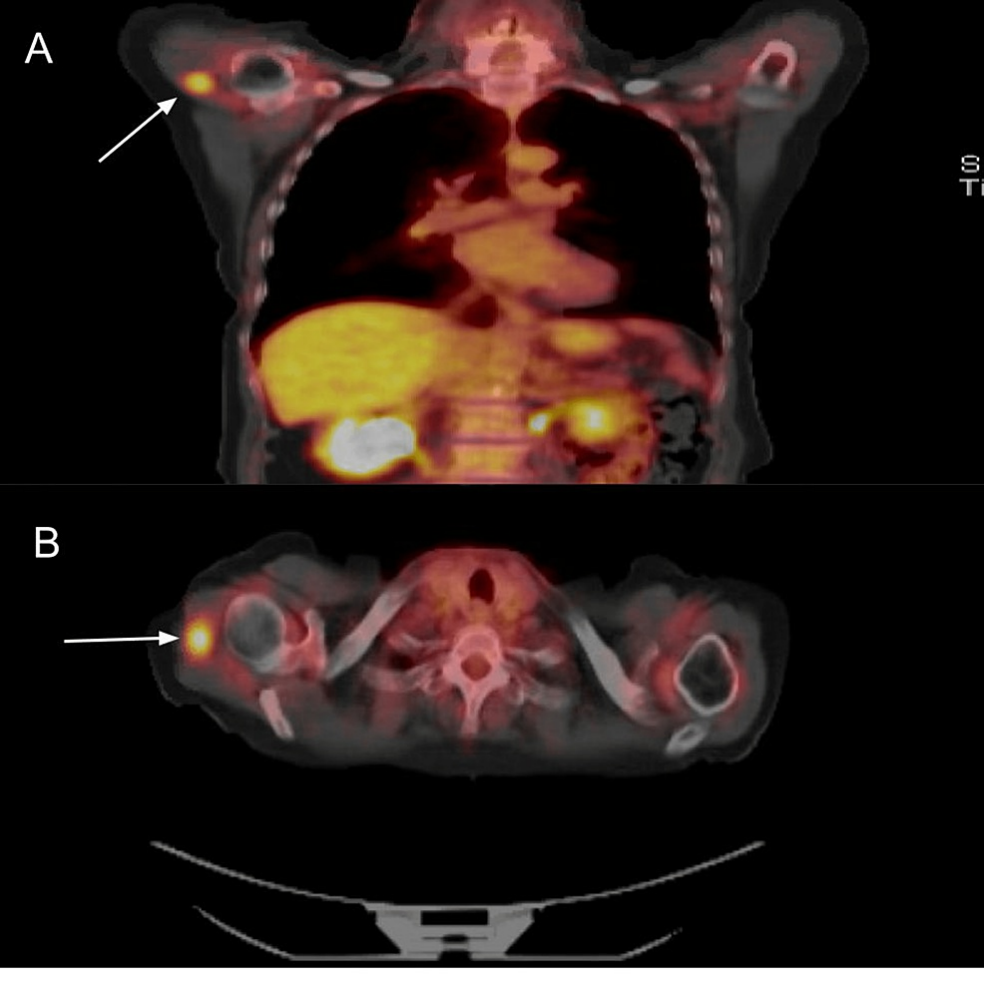
PET-CT showed an abnormal focal area of increased activity (white arrow) localizing to the proximal right deltoid muscle (A: coronal view, B: axial view). PET-CT: Positron emission tomography-computed tomography.

**Figure 7:**
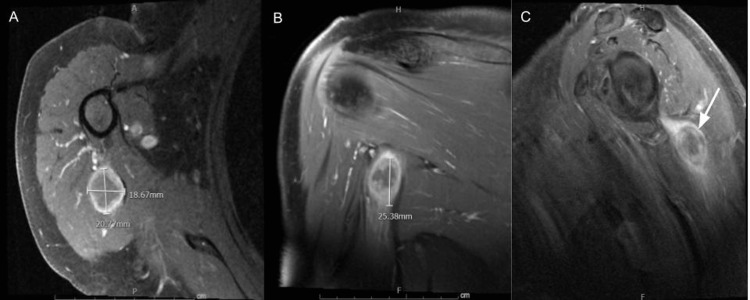
MRI of the right shoulder showed an enhanced solid mass (white arrow) within the proximal long head of the right tricep muscle (A: axial view, B: coronal view, C: sagittal view).

**Figure 8:**
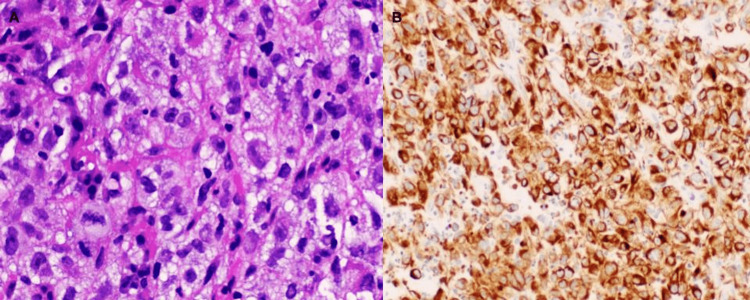
Right triceps, biopsy specimen: Poorly differentiated non-small cell carcinoma, histologically resembling patient's known lung primary. (A) Highly disorganized large cells with irregularly shaped nuclei and scant cytoplasm (H&E x200). (B) immunohistochemical positivity for CK 7 staining (Cytokeratin 7 x100).

Treatment options with surgical resection were discussed for her early-stage lung cancer, but she refused surgery and chose stereotactic body radiation therapy for ease of recovery. After radiation therapy, she was followed up with interval chest CT imaging. At 11 months of surveillance, the chest CT showed an interval increase in the right middle lobe nodule concerning tumor progression (Figure 4). Follow-up PET-CT revealed mild activity in the right middle lobe, correlated with the density noted on a recent CT scan (Figure 5). Although the area appeared slightly larger on the recent CT scan relative to the prior, it showed low-grade activity. It also showed an abnormal focal area of increased activity localizing to the proximal right deltoid muscle (Figure 6). Malignancy was suspected, and a follow-up MRI of the right shoulder with and without IV contrast for further evaluation was suggested. MRI of the right shoulder showed a 2.9 x 1.9 x 2.5 cm enhanced solid mass within the proximal long head of the right triceps muscle corresponding to the uptake area on a recent PET scan and was suspicious for a metastatic lesion (Figure 7). Ultrasound-guided biopsy of the right triceps returned non-small cell carcinoma histologically similar to the patient's known primary lung cancer (Figure 8). Brain MRI was negative for metastatic disease.

**Figure 9:**
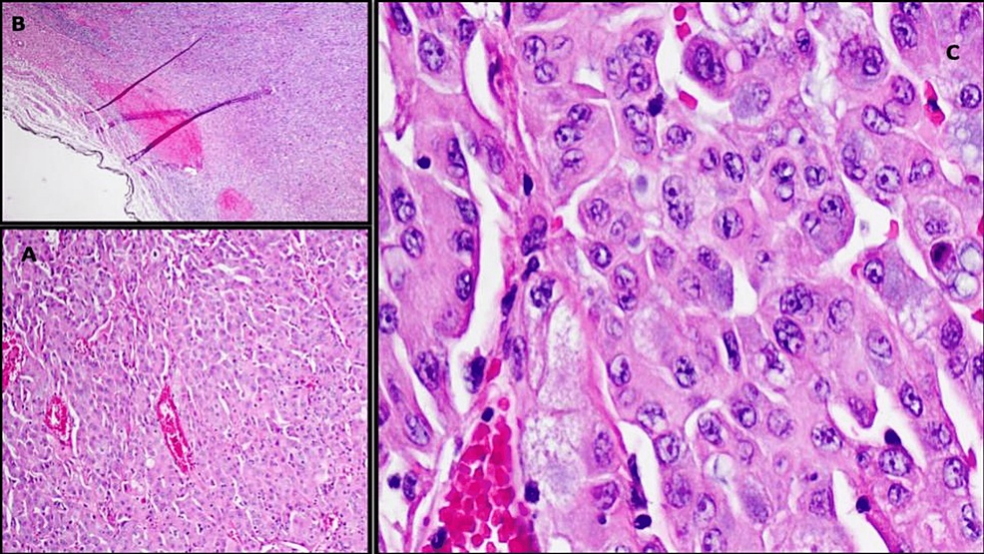
Right lung upper lobe, wedge resection showing (A: H&E, x200) invasive poorly differentiated adenocarcinoma (0.8 cm), with poorly differentiated cells with minimal glandular differentiation arranged in a solid growth pattern and demonstrating lymphovascular invasion. (B: H&E, x100) Invasive carcinoma is located less than 1 mm from the parenchymal surgical margin. (C: H&E, x100) Tumor cells appear pleomorphic, irregularly shaped, and packed in solid nests with features of prominent nucleoli, high nuclear-to-cytoplasmic ratio, and minimal cytoplasm.

**Figure 10:**
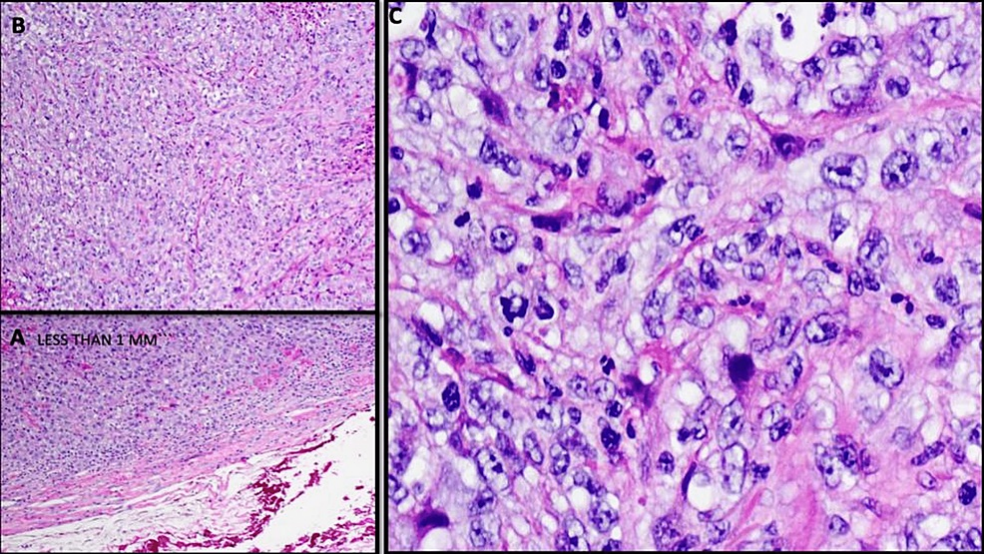
Histology of the long head of the right tricep muscle, resection demonstrating metastatic non-small cell carcinoma (5.5 cm tumor mass). A) Carcinoma is located less than 1 mm from medial and posterior surgical margins (H&E, x100). B) Neoplastic cells infiltrating between muscle fibers (H&E, x100). C) Foamy vacuolated cytoplasm, fine chromatin, and variable prominent nucleoli (H&E, 200x).

She underwent two wedge resections from the right middle and upper lobes of the lung and right triceps muscle tumor excision with reconstruction. Histology of right middle lobe wedge resection showed benign lung parenchyma, negative for malignancy; changes were consistent with post-radiation therapy. The right upper lobe wedge resection was positive for invasive, poorly differentiated adenocarcinoma with carcinoma located less than 10 mm from the parenchymal surgical margin; the lymphovascular invasion was present (Figure 9). The right tricep muscle resection pathology report revealed metastatic non-small cell carcinoma consistent with the patient's known primary lung cancer measuring 5.5 cm and extending less than 1 mm from the medial and posterior surgical margins (Figure 10). Her previous lung biopsy was TTF-1 negative, the same as the tricep muscle biopsy. Tumor cells in tricep muscle biopsy were positive for CAM 5.2 and Cytokeratin 7 (CK7) and negative for CK20, EMA, S100, and Mucicarmine. The tumor was GATA-3 negative. Tumor cells were 100% positive for programmed death-ligand 1 (PD-L1). Afterward, she underwent radiation therapy to the operative bed of the right tricep tumor excision, given the presence of close margins.

At the time of this writing, she is on adjuvant endocrine therapy with letrozole. She is closely followed up by multi specialties, including pulmonology, oncology, primary care provider, and breast surgery. She is routinely monitored with clinical breast exams twice yearly, annual mammography screening, and CT chest, abdomen, and pelvis every four months. Four years after excision and radiation therapy, she remains in remission.

## Discussion

Lung cancer has the second highest mortality rate among all cancers. The prevalence of NSCLC is about 80%-85% among all cancers, while that of small cell lung cancer is 10%-15% [3]. The relative five-year survival rate of localized NSCLC is 65%, and of all surveillance, epidemiology, and end results (SEER) stages combined is 28% [4]. It is known to have the potential for metastasis, and the most common sites are the brain, liver, and adrenals. However, metastasis of lung adenocarcinoma to skeletal muscle is very rare. Several factors contribute to this, including variable blood flow rates in muscles during exercise and rest, which reduce tumor cell adhesion to underlying tissues, the ability of skeletal muscles to produce interleukin-6 and leukemia inhibitory factor, as well as the ability of muscle microvasculature to destroy cancer cells and cause neovascularization through lactic acid [5]. ​​After stereotactic body radiation therapy for stage 1 adenocarcinoma of the lung, the patient underwent surveillance scans. During the surveillance CT scan 11 months after her initial diagnosis, metastasis was found in her trapezius muscle. Therefore, these patients must be closely monitored to detect cancer spread in their early stages and initiate treatment on time. Skeletal muscle metastasis is seen when it spreads to distant organs via blood or lymphatic pathways. Despite being richly supplied with vessels, metastasis rates in skeletal muscles are very low compared to other organs. There are several hypotheses for metastasis to skeletal muscles. The mechanisms of metastasis are thought to be via the blood vessels or lymphatic channels or by direct extension from adjacent structures. The most commonly accepted hypothesis supports the idea of spread via vasculature. It suggests that tumor embolism is the cause of metastasis in the muscles [6]. The most common sites of skeletal muscle metastasis of lung cancer are the thigh, iliopsoas, and paraspinous muscles. Lesions have also been reported in the orbit and pectoral muscles [7-10]. Besides, some literature also reports the calf muscles being among the common spreading sites [11]. Diagnosing metastasis to the skeletal muscles can be confusing as the symptoms can be misunderstood as muscle strain or injury. It usually presents as painful and palpable masses with or without localized swelling [8]. However, our patient was asymptomatic and was incidentally found to have tricep muscle metastasis in surveillance images. CT scans, MRIs, and PET scans help diagnose; however, a histopathological examination is needed to confirm the diagnosis. MRI features of metastasis in skeletal muscles can be classified into three categories. Type-I localized lesions with heterogeneous iso-signal intensity in T1-weighted image (T1WI) and heterogeneous hyperintensity in T2-weighted image (T2WI). Type-II diffuse lesions without bone destruction and are characterized by diffused swelling of muscle and slightly hypo- to iso-intensity in T1WI and hyperintensity in T2WI. Type-III diffuse lesions with bone destruction, characterized by irregular lumps with iso-intensity in T1WI and heterogeneous hyper-intensity in T2WI, with adjacent bone invasion [12].

Treatment plans are decided according to the extent of metastasis and the patient's level of functioning or performance status. Nevertheless, still, there are no optimal treatment strategies for skeletal metastasis of lung cancer. A surgical removal is an option for patients with good functional status and localized mass/oligo metastasis. Chemotherapy and radiation therapy are recommended for patients not surgically treated. The prognosis of NSCLC metastasized to skeletal muscles is uncertain, with a median survival period of only six months [13]. Generally, stage IV NSCLC is treated with chemotherapy [14]. The patient, however, underwent surgical resection because of her oligometastasis. Due to a close surgical margin of less than 1 mm, she received radiation therapy to the tricep muscle operative bed. A four-year follow-up has shown no evidence of further metastasis, and she is receiving adjunct endocrine therapy for her breast cancer. Given the unusual site of lung adenocarcinoma metastasis, this is a rare and interesting case. This patient remains disease-free for four years as most other reported cases of skeletal muscle disease have poor survival, not beyond one year.

## Conclusions

Trapezius muscle metastasis from lung adenocarcinoma is very rare. The optimal treatment plan for skeletal muscle metastasis is debatable. Treatment options are selected after assessing the patient's performance status and the extent of the metastasis. As in this case, the oligometastatic disease can be treated surgically. This case report highlights the importance of close surveillance of patients with carcinoma, as some metastases will not cause symptoms in the early stages. The patient in this case report was treated surgically and followed up with radiation therapy. Up to now, she has been disease-free for four years, while the other reported cases have poor survival.
